# Proximate and distal factors associated with the stall in the decline of adolescent pregnancy in Uganda

**DOI:** 10.1186/s12889-021-11403-6

**Published:** 2021-10-18

**Authors:** Catherine Mbabazi, Alexander Kintu, John Bosco Asiimwe, John S. Ssekamatte, Iqbal Shah, David Canning

**Affiliations:** 1 Department of Global Health and Population, Harvard T.H. Chan School of Public Health, Boston, MA USA; 2 National Population Council, Ministry of Finance, Planning and Economic Development, Statistics House, Plot 9, Colville Street, P.O. Box 2666, Kampala, Uganda; 3 School of Statistics and Planning, Makerere University, Kampala, Uganda; 4 National Planning Authority, Kampala, Uganda

**Keywords:** Adolescent pregnancy, Sexual debut, Education attainment, Fertility determinants, Uganda

## Abstract

**Background:**

Adolescent pregnancy in Uganda declined from 31% in 2000–01 to 25% in 2006 but thereafter stalled at 25% from 2006 to 2016. This paper investigates the factors associated with the recent stall in the rate of decline of adolescent pregnancy in Uganda.

**Methods:**

We used logistic regression models for 4 years (2000–01, 2006, 2011 and 2016) of data from the Uganda Demographic Health Survey to explore proximate and distal factors of adolescent pregnancy in Uganda. We carried out Blinder-Oaxaca decomposition models to explore the contributions of different factors in explaining the observed decline in adolescent pregnancy between 2001 and 2006, and the subsequent stall between 2006 and 2016.

**Results:**

We found that marriage among women aged 15–19 years, and early sexual debut, were strongly associated with adolescent pregnancy. These declined substantially between 2000 and 01 and 2006, leading to a decline in adolescent pregnancy. Their decline was in turn associated with rising levels of female education and household wealth. After 2006, education levels and household wealth gains stalled, with associated stalls in the decline of marriage among women aged 15–19 years and sexual debut, and a stall in the decline of adolescent pregnancy.

**Conclusions:**

The stall in the decline of adolescent pregnancies in Uganda was linked to a stall in the reduction of adolescent marriage, which in turn was associated with limited progress in female educational attainment between 2006 and 2016. We emphasize the need for a renewed focus on girl’s education and poverty reduction to reduce adolescent pregnancy in Uganda and subsequently improve health outcomes for adolescent girls.

**Supplementary Information:**

The online version contains supplementary material available at 10.1186/s12889-021-11403-6.

## Background

Adolescent pregnancy is a global concern because of its effect on education, maternal and child health outcomes [1]. Complications from pregnancy and childbirth are a leading cause of death among adolescent girls [2]. Each year, 21 million girls aged 15–19 years become pregnant in low- and middle-income countries, and 16 million give birth [3]. Despite global progress in reducing adolescent pregnancy, projections indicate that adolescent pregnancy will increase globally by 2030, and Sub-Saharan Africa (SSA) will have the highest rates of pregnancies occurring among adolescents [3].

Current estimates indicate that SSA has high rates of adolescent pregnancy estimated at 19.3% [4]. In the SSA region, East Africa has the highest prevalence which is estimated at 21.5% [4] but there are country variations. For instance, Uganda has a higher prevalence of adolescent pregnancy compared to Kenya whose adolescent pregnancy is at 18% [5]. In Uganda, in 2016, a quarter of females aged 15 to 19 years were either pregnant with their first child or had previously had a baby [6]. Adolescent pregnancy declined from 31% in 2000–01 to 25% in 2006 but thereafter stalled at 25% from 2006 to 2016 [6]. Previous studies have explored factors influencing adolescent pregnancy in Uganda [7–10]. These studies identified enablers and barriers to contraceptive use among adolescents and young people. There is however paucity of information on the stall in the decline of adolescent pregnancy in Uganda.

This paper investigates the factors associated with the recent stall in the decline of adolescent pregnancy in Uganda. Results from this analysis can be used to inform health and social policies aimed at reversing the stall in the decline of adolescent pregnancy in Uganda.

## Methods

Our conceptual framework is based on proximate determinants of fertility [11] and the framework for early adolescence [12]. The Bongaarts model distinguishes between proximate factors that determine pregnancy and the distal socio-economic forces that determine the proximate factors. Similarly, the framework for early adolescence proposes an ecological approach that provides the context that shapes adolescent health. These ecological factors in turn influence early sexual debut, marriage, and pregnancy. This led us to develop a model with two stages: ecological and socioeconomic forces effect marriage among women aged 15–19 years and early sexual debut, and these in turn are associated with adolescent pregnancy.

### Study population

**Fig. 1:**
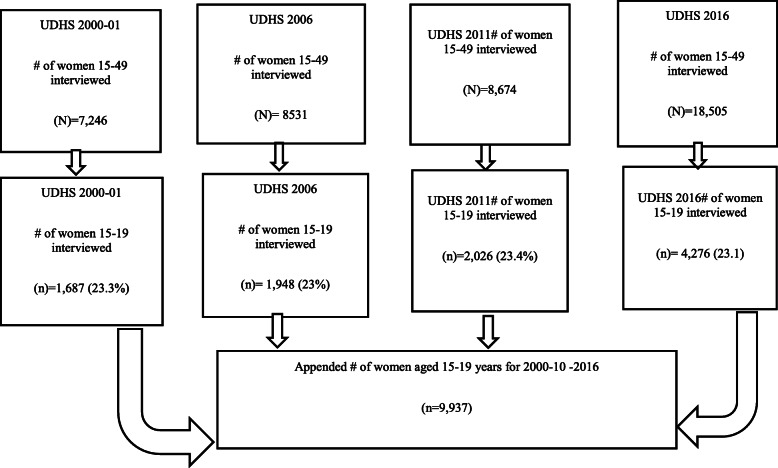
Derivation of study sample

We used secondary data from 4 years (2000–01, 2006, 2011 and 2016) of the Uganda Demographic Health Survey [6]. These nationally representative, repeated cross-sectional surveys collected demographic and health data for women aged 15–49. The surveys were conducted using a stratified two-stage cluster sampling process. Strata were urban and rural areas of regions, and clusters were randomly selected enumeration areas in each stratum. Within each cluster around 20 households were sampled. We used data for women aged 15–19 years old. We applied sampling weights to provide estimates that were representative of the population of Uganda at the time of each survey. The study sample was derived from the women 15–49 years individual recode file in the Uganda demographic health surveys including 4 years from 2000 to 01 to 2006. A total of 9937 women aged 15–19 years were interviewed. From the 2000/01 a total of 7246 women aged 15–49 years were interviewed and of those 1687 were 15–19 years. Similarly, in 2016 a total of 18,505 women aged 15–49 years were interviewed and of those 4276 were 15–19 years. Figure 1 demonstrates the detailed procedure followed in the selection of women 15–19 years from 2000 to 2016.

### Measures

#### Outcome

The outcome variable for this study was adolescent pregnancy among women 15–19 years old. This was defined as women aged 15–19 years old who have had a live birth or were pregnant with their first pregnancy at the time of the survey [13]. Those whose pregnancies did not end in a live birth were excluded from the analysis.

#### Proximate factors

The proximate factors we considered were sexual debut and ever married. Sexual debut was the age women reported first having sex [6]. Early sexual debut was defined as ever having had sex before the age of 15 years. The definition of early sexual debut was based on Blum’s framework for early adolescence defined as age 10–14 years [12]. We thus can categorize sexual debut as having occurred before, during, and after early adolescence. Ever married was defined as having been married or in union at the onset of pregnancy [6]. Marriage among women aged 15–19 years was defined as being married and ever in union among women 15–19 years. Although contraception and abortion are important proximate determinants of pregnancy, we dropped the variables; “Current use” and “Ever use” of contraception because of the nature of the DHS data, we were unable to determine timing of contraceptive use against that of pregnancy. Therefore, current use and ever use of contraception to control pregnancy was dropped because the timing of contraceptive use may have been after the result of the first pregnancy. It is highly likely that a significant number of adolescents may get pregnant with the first child but then use contraceptives to prevent the next pregnancy [14, 15]. This may lead to observations may indicate that contraceptive use increases the risk of adolescent pregnancy which may be counter-productive on messaging for uptake of modern contraceptives. Abortion was left out because previous UDHS have not measured abortion. Abortion data is also limited to post abortion care.

#### Distal factors

The distal variables are the respondent’s education level, wealth index, urban or rural residence, and region. Education is measured as a categorical variable with levels corresponding to no education, primary school education, and secondary education and above. Uganda operates a system of education with 7 years of primary education, 4 years of ordinary level secondary education and 2 years of advanced level secondary education and 2 to 5 years of tertiary education. Based on this system, the number of years spent in education is computed and this can be used to generate descriptive summaries. The asset indices reported in the demographic and health survey (DHS) are calculated differently in each survey, and while they allow comparisons across households within a survey, they do not allow direct comparison across surveys. To overcome this, we used Principal Component Analysis to calculate a common asset index across the four survey years using ownership of a set of household goods and services [16]. The variables that we used to create the wealth index included: main source of drinking water for members of the household, time taken to get to the water source for drinking water, type of toilet facility in the household, whether the household had electricity, whether the household has radio, whether the household has television, whether the household has refrigerator, whether any member of the household has bicycle, whether any member of the household has motorcycle/scooter, whether any member of the household has car/truck, main material for the floor, main material of the walls, main material of the roof, whether the household has a telephone, whether the household shared toilet facility with other households, and type of cooking fuel used by the household. We then allocated households to wealth quintiles based on their ranking on the asset index. Our approach allows us to take account of improvement over time in household ownership of assets and wealth.

### Statistical methods

We hypothesized that sexual debut and marriage are the proximate factors that affect adolescent pregnancy. We further hypothesized that family income and the girl’s education are the distal factors that are associated with sexual debut and marriage among women aged 15–19 years. We estimated models of the proximate determinants of adolescent pregnancy. We then constructed models of the more distal socioeconomic factors that affect sexual debut and marriage among women aged 15–19 years. We also estimated the reduced form of the total effect of the socioeconomic factors on adolescent pregnancy. The models are estimated over the entire study period, allowing for time trends. Regression Model 1: Proximate factors associated with adolescent pregnancy; Regression model 2: Distal factors associated with sexual debut; Regression model 3: Distal factors associated with marriage among women aged 15–19 years and Regression model 4: Distal factors associated with adolescent pregnancy.

We used the model estimates to perform a Blinder-Oaxaca decomposition [17] to estimate which factors explained the observed decline in adolescent pregnancy between 2001 and 2006, and the stall between 2006 and 2016. The decomposition explained the observed changes in adolescent pregnancy through changes in the associated proximate and more distal socio-economic determinants. Each of our four models involved a binary outcome (adolescent pregnancy or not). We estimated logistic models of the form;

$$ {\log}_e\left[\frac{p_i}{1-{p}_i}\right]=\alpha +{\beta}_1{x}_1+{\beta}_2{x}_2\kern0.5em +\dots .+{\beta}_n{x}_n $$

where; *p*_*i*_= Probability of the outcome, *α*= Constant, *β*= The regression parameter, and *xi* = are the predictor variables (for *i* =1, … ., *n*). We reported exponentiated coefficients which can be interpreted as odds ratios. Confidence intervals were calculated based on robust standard errors clustered at the survey cluster level. Observations with missing data for any variable were excluded from the analysis.

The Blinder Oaxaca linear decomposition can be used to explain changes in an outcome over time. Suppose we have a linear probability model:

$$ {y}_{it}={\beta}_1{x}_{it}+{\beta}_2{x}_{it}+{\beta}_n{x}_{nit}+\dots +{\gamma}_t+{\epsilon}_{it} $$

Where *y* = Dependent variable (adolescent pregnancy), *i* =individuals, *t* =Year of survey, *y*_*it*_ = observation for individual *y* at time *t*. *x* represents a predictor variable, *β* represents regression coefficients, and *γ*_*t*_ are survey year dummies. The decomposition of the expected change in the average outcome between year *t* and year *t* + *k* can be expressed as:

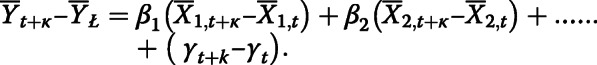



Where the variables $\overline{Y}$ and $\overline{X}$ denote the average outcome at the time of the survey. Note that by construction the average of the errors terms is zero given we have survey dummies.

The decomposition explores the contribution of selected variables to the mean outcome difference. The difference is divided into a part that is “explained” by differences in characteristics and a residual part that cannot be accounted for (the unexplained part) by differences in such characteristics. The explained part of the decomposition was the effect of the changes in the explanatory variables, multiplied by their coefficients. We used a linear model for the decomposition since the logistic model is multiplicative and its effects depend on interactions between changes in variables which are difficult to interpret. *Model 1: Predicted mean difference for Adolescent Pregnancy and Proximate Model & Year; Model 2: Predicted mean difference for Sexual Debut and Distal factors & Year; Model 3: Predicted mean difference for Marriage among women aged 15–19 years and distal determinants & Year Model and Model 4: Predicted mean difference for Adolescent Pregnancy and distal factors & Year.* All analyses were performed using Stata version 15 (Stata Corp., TX, USA).

### Ethical considerations

The study was approved by the ethical review board at the Harvard Chan School of Public Health and the use of the required dataset was approved by the DHS Program.

## Results

Table 1 shows the descriptive statistics for the women in our sample. The 2016 survey was larger than the earlier surveys and contributes a large fraction of our sample. The average age of women in our sample was 16.9 years. Twenty five percent were married at the time of the surveys and just under half had previously had sex. About a quarter were or had been pregnant. Few (4.6%) women had no schooling. Almost two thirds (64.8%) had only primary school level education with a minority having secondary or higher level. On average women had 6.2 years of schooling and by construction, 20% of the sample were in each wealth quintile. About three quarters of women lived in rural areas. We also reported age at first sex, age at marriage and age at first birth for women reporting these events (Supplementary Table S1).

**Table 1:** Characteristics of sample of women included in the analysis (weighted)

Characteristic	Survey year			
	2000/01 (*n* = 1615)	2006 (*n* = 1936)	2011 (*n* = 2048)	2016 (*n* = 4264)
**Age**	Percentage	Percentage	Percentage	Percentage
15	18.6	24.1	23.5	20.4
16	21.0	21.2	20.2	22.7
17	19.0	17.9	17.9	18.6
18	23.5	19.6	20.4	20.0
19	18.0	17.2	18.1	18.4
**Sexual debut**				
Never had sex	47.9	57.0	54.9	54.3
Less than 15 years	14.3	11.9	12.4	10.3
15–19 years	37.8	31.2	32.7	35.3
**Age at first marriage**				
Never married	67.7	77.6	77.3	77.2
Married aged < 15 years	6.7	3.0	3.2	2.8
Married aged 15–19 years	25.6	19.4	19.6	20.0
**Age at first birth**				
Never had a birth	74.4	80.8	81.9	80.6
Had birth aged< 1 year	2.4	1.5	1.7	1.2
Had birth aged 15–19 years	23.2	17.8	16.4	18.1
**Adolescent pregnancy**				
Never had a pregnancy	68.6	75.1	76.2	75.2
Had a pregnancy	31.4	24.9	23.8	24.8
**Residence**				
Urban	19.4	17.7	19.3	24.3
Rural	80.6	82.3	80.7	75.8
**Education attained**				
No education	9.1	3.5	2.9	1.8
Primary	66.1	66.7	64.8	64.7
Secondary+	24.8	29.8	32.3	33.5
**Wealth quintile**				
Lowest	18.9	15.3	15.4	17.9
Second	18.7	17.5	16.9	19.7
Middle	15.9	17.3	18.0	19.1
Fourth	14.8	20.2	23.5	20.0
Highest	31.7	29.8	26.2	23.2
**Region**				
Central	35.7	30.0	30.2	26.6
Eastern	24.8	22.5	25.4	37.1
Northern	16.1	19.6	18.2	13.3
Western	23.4	27.9	26.2	23.0

**Fig. 2:**
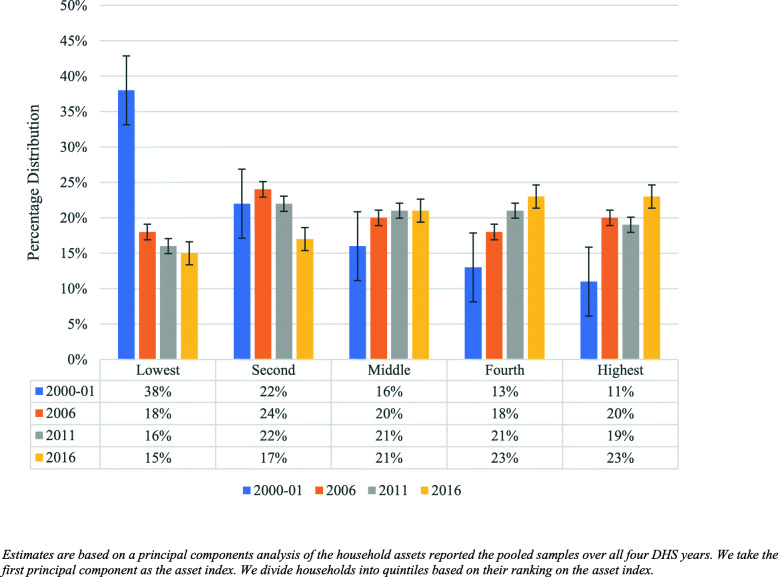
Wealth Index by Year: UDHS 2000–01 -2016

Figure 2 shows the evolution of our wealth index across years. In 2000–01, 37% of households were in the lowest wealth quintile as measured by the asset index. This falls substantially and by 2006, just over one fifth are in the lowest quintile. In the later period’s households tended to get a little wealthier but the gains were not as large as we see in the first period between 2000/01 and 2006. The percentage of the population in the lowest wealth quintile dropped from 37% in 2000–01 to 11% in 2016.

The results in Table 2 show that more than half (61% in 2001, 59% in 2006, 58% in 2011 and 54% in 2016) of adolescent women aged 19 years had ever been pregnant. Also, 54, 41, 37 and 40% of adolescent women who were aged 18 years in the 2000/01, 2006, 2011 and 2016 surveys respectively, had ever been pregnant. Table 2 shows more details. The results also show that from 2001 to 2011, the adolescent pregnancy among women whose sexual debut was younger than 15 years reduced from 70% in 2001 to 53% in 2006 and 50% in 2011. There was however an increase in 2016 as the percentage of such women was 57%. The results also indicate that among women whose reported sexual debut was 15–19 years, the proportion that had ever been pregnant was respectively 57, 60, 54 and 54% in the 2000/01, 2006, 2011 and 2016 survey. The results also show that the proportion of never married adolescents who had ever been pregnant was 6% in 2000/01, 7% in 2006, 6% in 2011 and 8% in 2016. Also, among the adolescent women whose age at first marriage was younger than 15 years, most (97% in 2000/01, 96% in 2006, and in 2011 and 93% in 2016) had ever been pregnant. Similarly, majority (81% in 2000/01, 86% in 2006, 82% in 2011 and 2016) of the adolescent women whose age at first marriage was 15–19 years had ever been pregnant. Also, the study shows that 26% of women who had never used contraceptives in the 2000/01 survey had ever been pregnant. This percentage was 18% in 2006, 19% in 2011 and 17% in 2016. On the other hand, the results indicate that majority (72% in 2000/01, 77% in 2006, 53% in 2011 and 58% in 2016) of adolescent women who said they had ever used contraceptives had also ever been pregnant.

**Table 2:** Adolescent pregnancy by selected characteristics

Characteristic	Ever been pregnant							
	2001		2006		2011		2016	
	No (%)	Yes (%)	No (%)	Yes (%)	No (%)	Yes (%)	No (%)	Yes (%)
**Age**								
15	96.7	3.3	98.1	1.9	98.4	1.6	96.9	3.1
16	87.1	12.9	91.6	8.5	91.5	8.5	90.7	9.4
17	76.8	23.2	74.6	25.5	79.2	20.8	77.9	22.1
18	46.0	54.0	59.0	41.0	62.6	37.4	59.8	40.2
19	38.8	61.2	41.4	58.6	42.4	57.6	46.1	53.9
**Sexual debut**								
Less than 15 years	29.8	70.2	47.3	52.7	50.0	50.0	43.3	56.7
15–19 years	43.4	56.6	40.2	59.9	46.1	54.0	46.4	53.6
**Age at first marriage**								
Never married	93.6	6.4	93.0	7.0	93.9	6.1	92.5	7.5
Married aged younger than 15 years	3.1	96.9	4.4	95.6	4.5	95.5	7.1	92.9
Married aged 15–19 years	19.5	80.5	14.2	85.8	17.9	82.1	17.8	82.2
**Contraception**								
No	74.4	25.6	82.2	17.8	81.1	18.9	82.6	17.4
Yes	28.5	71.5	22.9	77.1	47.0	53.0	42.4	57.6
**Residence**								
Urban	77.5	22.6	80.3	19.7	78.6	21.4	81.2	18.8
Rural	66.5	33.6	74.0	26.1	75.6	24.4	73.3	26.7
**Education attained**								
No education	41.0	59.0	49.8	50.2	55.5	44.5	65.4	34.6
Primary	66.9	33.1	72.1	27.9	73.1	26.9	71.3	28.7
Secondary+	83.3	16.7	84.7	15.3	84.2	15.8	83.3	16.7
**Wealth quintile**								
Lowest	63.0	37.0	59.3	40.7	65.6	34.4	66.5	33.5
Second	75.9	24.1	68.8	31.2	67.2	32.9	68.1	31.9
Middle	70.2	29.8	77.8	22.2	75.7	24.3	75.5	24.6
Fourth	73.9	26.1	77.4	22.6	80.9	19.1	78.5	21.5
Highest	64.3	35.7	83.7	16.3	84.2	15.8	84.9	15.1
**Region**								
Central	68.8	31.2	78.2	21.8	79.0	21.0	76.9	23.1
Eastern	63.5	36.5	71.8	28.2	69.6	30.4	73.1	27.0
Northern	65.7	34.3	69.5	30.5	73.4	26.6	77.0	23.0
Western	75.7	24.3	78.3	21.7	81.1	18.9	75.7	24.3

The findings in Table 2 indicate that the proportion of adolescent women from rural areas who had ever been pregnant reduced from 34% in the 2000/01 survey to 26% in 2006 to 24% in 2011 but this rose to 27% in 2016. Also, the proportion of adolescent women from urban areas who had ever been pregnant reduced from 23% in 2000/01 to 20% in 2006 but this slightly increased to 21% in 2011 and then reduced to 19% in 2016. The results show that the proportion of adolescent women from the lowest quintile who had ever been pregnant was 37% in 2000/01, 41% in 2006, 34% in 2011 and 2016%. Also, 24, 31, 33 and 32% of adolescent women from the second lowest wealth quintile in 2000/01, 2006, 2011 and 2016 respectively had ever been pregnant. The results also indicate that among the adolescent women from the highest quintile, 36%, in 2000/01, 16% in 2006 and 2011, and 15% in 2016 had ever been pregnant. The results in the Table also show that in the 2000/01 survey, 31% of adolescent women from central region, 36% of those from the eastern, 34% of those from Northern and 24% of those from western had ever been pregnant. Also the results indicate the proportion of adolescent women from Central region who had ever been pregnant 22% in 2006, 21% in 2011 and 23% in 2016. Additionally, 28, 30 and 27% of adolescent women from the Eastern region in 2006, 2011 and 2016 surveys respectively had ever been pregnant. The results further show that 30, 27, and 23% of adolescent women from the Northern region in the 2006, 2011 and 2016 surveys respectively had ever been pregnant. Also, the findings indicate that proportion of women from the Western region who had ever been pregnant was 22, 19 and 24% in the 2006, 2011 and 2016 surveys respectively.

Table 3 shows the results of our logistic regressions. In Model 1, we evaluated proximate determinants of adolescent pregnancy in our sample. We controlled for age at the time of interview and for survey year effects, but none of the year effects were statistically significant. Women who had never had sex were not at risk of pregnancy and were dropped from the logistic regression, since they were perfectly predicted as not having a pregnancy. The reference group for the regression was therefore those who have ever had sex. We found that adolescent girls who initiated sex below the age of 15 years had almost double the odds of having a teenage pregnancy when compared to those who reported sexual debut after age 15 (Odd Ratio [OR]: 1.85, 95% C.I: 1.51,2.28). Married adolescents also had much higher odds (OR: 13.76) of experiencing a pregnancy than unmarried ones. Older adolescent girls were more likely to report pregnancy since they had a longer period of exposure to becoming pregnant. While we had data on current use of contraception, another proximate determinant, we did not use it in Model 1 because this was measured at the time of interview, and after any pregnancy has occurred. If a woman is currently pregnant, she will not be using contraception, but the causality runs from the pregnancy to the lack of current family planning use not vice versa. Again, pregnancy prior to the interview date will depend on past, not current, contraceptive use.

**Table 3:** Factors associated with adolescent pregnancy in Uganda, Demographic and Heath Surveys 2000–2016

	Regression Model 1	Regression Model 2	Regression Model 3	Regression Model 4
Outcome	Adolescent pregnancy	Sexual debut	Marriage among women aged 15–19 years	Adolescent pregnancy
**Year**				
2000–01	Ref	ref	ref	ref
2006	1.26 [0.95,1.66]	0.85 [0.69,1.06]	0.71^*^ [0.54,0.93]	0.92 [0.72,1.16]
2011	0.92 [0.70,1.22]	0.89 [0.71,1.11]	0.72^*^ [0.54,0.95]	0.82 [0.63,1.06]
2016	1.04 [0.81,1.34]	0.86 [0.71,1.04]	0.77^*^ [0.60,0.97]	0.93 [0.75,1.15]
**Sexual Debut**				
15–19 years	Ref			
14 years or less	1.85^***^ [1.51,2.28]			
**Marital Status**				
Never married	Ref			
Ever married	13.76^***^[11.49,16.47]			
**Age**				
Age	1.58^***^ [1.45,1.73]	2.22^***^[2.12,2.33]	2.81^***^[2.67,2.97]	2.74^***^ [2.59,2.90]
**Education Level**				
No education		ref	ref	ref
Primary		0.53^***^[0.36,0.76]	0.33^***^ [0.22,0.48]	0.47^***^ [0.31,0.70]
Secondary+		0.29^***^ [0.20,0.42]	0.07^***^ [0.05,0.11]	0.13^***^ [0.08,0.19]
**Wealth Index**				
Lowest		ref	ref	ref
Second		0.91 [0.77,1.09]	0.93 [0.76,1.14]	0.89 [0.73,1.08]
Middle		0.94 [0.78,1.13]	0.69^**^ [0.56,0.86]	0.75^**^ [0.60,0.93]
Fourth		0.94 [0.76,1.16]	0.70^**^ [0.55,0.88]	0.66^***^ [0.52,0.85]
Highest		0.81^*^ [0.66,1.00]	0.58^***^ [0.45,0.76]	0.48^***^ [0.37,0.63]
**Country Region**				
Central		ref	ref	ref
Eastern		1.08 [0.91,1.29]	1.17 [0.93,1.48]	1.00 [0.81,1.23]
Northern		0.66^***^ [0.52,0.84]	0.88 [0.67,1.16]	0.71^**^ [0.55,0.92]
Western		0.57^***^ [0.47,0.69]	0.68^**^ [0.53,0.86]	0.58^***^ [0.46,0.73]
**Place of residence**				
Urban		ref	ref	ref
Rural		0.96 [0.82,1.13]	1.16 [0.94,1.44]	1.10 [0.90,1.35]
**Sample size (#obs)**	**4450**	**9009**	**9009**	**9009**

Coefficients are reported as odds ratios [95% C.I] in brackets

Regression Model 1: Proximate factors of adolescent pregnancy; Regression Model; 2: Distal factors of sexual debut; Regression Model; 3: Distal factors of marriage among women aged 15–19 years and Regression Model 4: Distal factors of Adolescent pregnancy

^*^ *p* < 0.05, ^**^ *p* < 0.01, ^***^ *p* < 0.001

Models 2 and 3 of Table 3 show results for associations between socioeconomic factors and our proximate determinants of teenage pregnancy. Model 2 shows the socioeconomic factors and sexual debut. We found no significant survey year effects, but age was strongly associated with sexual debut – each added year of age doubled the estimated odds of sexual debut (OR: 2.22). A higher level of education, and being in a richer wealth quintile, were strongly associated with lower risk of sexual debut. Compared to those with no education, adolescents with a primary school education had lower odds (OR: 0.53) of sexual debut, and those with a secondary school level of education even lower odds (OR: 0.29). The gradient in wealth quintile was not so steep, we found a significant association only in the highest wealth quintile compared to the lowest. We also found significant regional associations; adolescent girls in the Western and Northern Uganda had lower odds of sexual debut compared to other regions, which may reflect regional differences in cultural beliefs and traditions regarding restrictions on premarital sex and early marriage [2, 18–23]. However, we found no significant effect of rural versus urban residence.

Model 3 shows results for associations between socioeconomic factors and marriage (marriage among adolescents aged 15–19 years). As expected, the likelihood of being married rises with the adolescents’ age. We found strong gradients with education and the wealth quintile; educated girls, and those in richer households, were much less likely to be married. Marriage among women aged 15–19 years is less common in the Western region, but again we found no significant difference between urban and rural residence. However, a striking feature of Model 3 was that relative to 2000–01 the subsequent years found decline in marriage among women aged 15–19 years due to survey year effects. Marriage among women aged 15–19 years falls for unexplained reasons in 2006, and then remains at this lower level.

We can think of the socioeconomic factors, such as education, wealth quintile, and place of residence, acting on the proximate factors, sexual debut and marriage among women aged 15–19 years, and these in turn influencing adolescent pregnancy. In model 4 of Table 3, we show a model explaining adolescent pregnancy with the distal socioeconomic factors. In this model we did not control for the mediating proximate factors since we wanted to assess distal associations. Note that the outcome changes slightly relative to Model 1 due to the different sample when we added covariates. This model found significant associations for the girl’s age, and lower risks of adolescent pregnancy with higher education levels and wealth quintiles, as well as region effects.

We now turn to our decomposition analysis shown in Table 4. The top portion of Table explains the changes observed over the period 2000–01 to 2006 while the bottom portion explains the changes over the period 2006–2016. Model 1 examined the role of the proximate determinants in contributing to the change in adolescent pregnancy. Adolescent pregnancy declined from 31% in 2000–01 to 25% in 2006, a drop of 6%. Delayed marriage and sexual debut were the proximate factors associated with the decline. Our sample in 2006 was a little younger than in 2000–01 and this change in age structure explains a further 1% decline in reported pregnancies. The large declines in sexual debut and marriage among women aged 15–19 years over the period 2001–2006 were examined in Models 2 and 3 of Table 4. To some extent these declines were due to the sample being younger in the later period, however there were also significant contributions from rising education levels and household wealth. In our model of the distal factors in Model 4 of Table 3, we can also see that the explanation of the decline in adolescent pregnancy was partially a change in the age structure of the sample, but there were also significant contributions of education levels and household wealth.

**Table 4:** Blinder-Oaxaca Decomposition Results. Estimated changes in adolescent pregnancy trends and attributable characteristics, Uganda Demographic and Heath Surveys 2000–2016

	Model 1	Model 2	Model 3	Model 4
Outcome	Adolescent Pregnancy	Sexual Debut	Marriage among women aged 15–19 years	Adolescent Pregnancy
**Change 2001–2006**				
2000–01	0.31^***^ [0.29,0.34]	0.52^***^ [0.49,0.55]	0.32^***^ [0.28,0.35]	0.31^***^ [0.28,0.34]
2006	0.25^***^ [0.23,0.27]	0.43^***^ [0.41,0.46]	0.22^***^ [0.19,0.24]	0.24^***^ [0.22,0.27]
Difference	0.06^***^ [0.03,0.10]	0.09^***^ [0.04,0.13]	0.10^***^ [0.06,0.14]	0.07^***^ [0.03,0.11]
Explained	0.08^***^ [0.05,0.11]	0.06^***^ [0.04,0.09]	0.07^***^ [0.04,0.09]	0.07^***^ [0.05,0.10]
**Contribution of explanatory variables to explained difference**				
Sexual Debut	0.02^***^ [0.01,0.03]			
Marital Status	0.06^***^ [0.03,0.08]			
Age	0.01^**^ [0.00,0.01]	0.03^**^ [0.01,0.05]	0.02^** [^0.01,0.04]	0.03^**^ [0.01,0.04]
Education		0.02^***^ [0.01,0.02]	0.03^***^ [0.01,0.04]	0.02^***^ [0.01,0.03]
Wealth		0.01 [−0.00,0.02]	0.01^**^ [0.01,0.02]	0.02^**^ [0.01,0.03]
Regional variation		0.01^**^ [0.00,0.02]	0.01[− 0.00,0.01]	0.01^*^ [0.00,0.02]
Rural		0.00 [−0.00,0.00]	−0.00 [− 0.00,0.00]	−0.00 [− 0.00,0.00]
**Sample size (#obs)**	**3635**	**3175**	**3175**	**3175**
**Change 2006–2016**				
2006	0.25^***^ [0.23,0.27]	0.43^***^ [0.41,0.46]	0.22^***^ [0.19,0.24]	0.24^***^ [0.22,0.27]
2016	0.25^***^ [0.23,0.26]	0.45^***^ [0.43,0.47]	0.22^***^ [0.20,0.24]	0.24^***^ [0.23,0.26]
Difference	0.00 [−0.03,0.03]	−0.02 [− 0.06,0.02]	−0.00 [− 0.03,0.02]	0.00 [− 0.03,0.03]
Explained	−0.01 [− 0.03,0.01]	−0.02^*^ [− 0.04, − 0.00]	0.00 [− 0.01,0.02]	−0.00 [− 0.02,0.02]
**Contribution of explanatory variables to explained difference**				
Sexual Debut	−0.01 [− 0.01,0.00]			
Marital Status	−0.00 [− 0.02,0.01]			
Age	−0.00 [− 0.00,0.00]	−0.01 [− 0.03,0.00]	−0.01 [− 0.02,0.00]	−0.01 [− 0.02,0.00]
Education		0.00 [−0.00,0.01]	0.01[− 0.00,0.02]	0.01 [− 0.00,0.01]
Wealth		0.00 [−0.00,0.00]	0.01^*^ [0.00,0.01]	0.01^*^ [0.00,0.01]
Regional variation		−0.01^***^ [− 0.02, − 0.01]	−0.00 [− 0.01,0.00]	−0.00 [− 0.01,0.00]
Rural		0.00 [−0.00,0.00]	0.00 [− 0.00,0.00]	0.00 [− 0.00,0.00]
**Sample size (#obs)**	**5653**	**5983**	**5983**	**5753**

95% confidence intervals in brackets ^*^ *p* < 0.05, ^**^ *p* < 0.01, ^***^ *p* < 0.001

Categorical variables

Education level: no education primary secondary+

Regional variation: central eastern northern western

Wealth Index: Lowest_1 Second_2 Middle_3 Fourth_4 Highest_5

Estimates and confidence intervals are based on DHS samples adjusted for stratification and sampling weights to make the averages representative of the population

Model 1: Predicted mean difference for Adolescent Pregnancy and Proximate Model & Year; 2: Predicted mean difference for Sexual Debut and Distal factors & Year; Model 3: Predicted mean difference for Marriage among women aged 15–19 years and distal determinants & Year Model and 4: Predicted mean difference for Adolescent Pregnancy and distal factors & Year

In the lower portion of Table 4 we repeated the decomposition analysis for the period 2006–2016. Adolescent pregnancy went up, but only slightly, over this period. Our decomposition results in Table 4 suggest there was a small rise in the proportion of girls reporting delayed sexual debut over this period, but all the distal explanatory variables associated with adolescent pregnancy were remarkably stable and unchanging.

## Discussion

This study focused on understanding the factors associated with change in the rate of decline and stalled decline of adolescent pregnancy in Uganda from 2006 to 2016. Despite a considerable decline in the percentage of girls age 15–19 years who had given birth or were pregnant with their first child between 2000 and 01 and 2006, the rate of decline stagnated between 2006 and 2016 with one quarter of adolescent girls surveyed reporting a pregnancy.

We found that marriage and early sexual debut were strongly associated with adolescent pregnancy. These declined substantially between 2000 and 01 and 2006 leading to a decline in adolescent pregnancy. The results of our study are consistent with other studies [18, 23–26]. This decline in the proximate determinants of adolescent pregnancy was in turn associated with rising education levels and wealth, over the time period. These findings are again consistent with numerous studies [2, 18, 19, 21, 22, 24–34]. After 2006 there was very limited improvement in girl’s education, and household wealth, and a slight rise in sexual debut and adolescent pregnancies.

Our results suggest that the stall in the decline of adolescent pregnancies in Uganda from 2006 to 2016 can be traced back to a stall in the decline of marriage among women aged 15–19 years and early sexual debut, which in turn were associated with a stagnation in girls’ education levels and household wealth. This suggests a focus on girls’ education as a policy to reduce adolescent pregnancy, though it leaves open the policy question of whether more targeted interventions on the proximate determinants could succeed. Even after the introduction of Universal Secondary Education program in Uganda, national statistics indicate that the share of girls who complete at least a secondary level of education remains low [35] and a significant proportion of women (23–24%) are illiterate [36]. It has also been found that in the 2011–2016 period, the proportion of children that have never been to school slightly increased [37]. Also, there were lower rates of secondary school attendance which were linked to challenges such as child labor, teenage pregnancy and early marriage among girls that lead to school dropout in Uganda [37]. It has been noted that the completion of primary and transition to secondary level of education have favored children from better socio-economic backgrounds and urban areas [38].

Regions such as the north and east parts of Uganda have gone through periods of insecurity and thus many children including girls did not enroll in school. Moreover, in the central region that has large cities and in the western region of the country, urban life styles, peer pressure and activities that produce quick gains lead to demotivation among families that may partly explain the slow progress in completion of education [37].

### Strength and limitations of the study

The study’s strengths include the use of individuals level data from four rounds of the Uganda DHS. This study used national representative data for the periods 2000–2001 up to 2016 to explore the major factors contributing to the stall in the declining in adolescent pregnancy in Uganda. The current study is among the few to have explored a stall in the reduction of adolescent pregnancy using an approach that quantifies the contribution of selected factors to this stall. Furthermore, the study was based on theoretical models of Bongaarts’ framework on proximate determinants of fertility from the 1970s and Blum’s framework for early adolescence from 2014. These theoretical frameworks formed the criteria for selecting distal and proximate factors driving decline in adolescent pregnancy from 2001 and 2006 and the stall between 2006 and 2016. The theoretical frameworks are among those that are highly regarded in explaining proximal and distal determinants of fertility. However, due to the cross-sectional nature of DHS data, we were unable to establish causal relations. Therefore, the study is limited to describing the associations of the risk factors and stalled adolescent pregnancy in Uganda. The study was also limited by the availability of variables in the DHS that are key to exploring adolescent pregnancy, as these variables were missing. Distal factors measured at the time of interview may not reflect the causal factors in place at the time the woman became pregnant. Results from Uganda demographic and health survey reports of 2000/01, 2006, 2011 and 2016 indicated that the use of modern contraceptives among adolescent women was estimated at 9.0% in 2000/01, 5.2% in 2006, 6.0% in 2011 and 9.4% in 2016 [39–41]. Clearly, there is an indication of a stall in the use of modern contraceptive methods which could be associated with the stall in the decline of adolescent pregnancy during the 2000–2016 period. Use of contraception to control pregnancy may be an important proximate determinant but was not included in the analysis because the timing of contraceptive use may have been after getting pregnant and thus the interpretation of the findings may be misleading due to reverse causality. Prior adolescent pregnancy may increase the likelihood of using contraceptives to prevent another pregnancy.

Our analysis likely underestimated the number of adolescent pregnancies because the outcome variable excludes pregnancies that did not end into a live birth. In the DHS, teenage pregnancy/motherhood is defined as the percentages of teenage girls (15–19 years) who are mothers, pregnant with their first child, and have begun childbearing at the time of the surveys. The estimation looks at the Percentage of women who have begun childbearing is the sum of the percentage who are mothers and the percentage who are pregnant with their first child. This excludes pregnancies that didn’t end into a live birth. Also, the analysis does not control for contraception use and abortion which are known proximate determinants of pregnancy/fertility. Abortion data in the DHS are also limited to post abortion care.

## Conclusion

The stall in the decline of adolescent pregnancies in Uganda was linked to a stall in the reduction of marriage among women aged 15–19 years, which in turn was associated with limited progress in female educational attainment between 2006 and 2016. Increasing years of schooling for women to 13 years or more is one potential strategy to address the stall in adolescent pregnancies in Uganda. In addition, encourage families to keep girls in school through community activism and enforcement of policies and laws on Universal Primary and Secondary Education, age at marriage and ending all harmful practices against women including gender-based violence. Our results suggest the need for a renewed commitment to girl’s education in Uganda, as a mechanism for reducing adolescent pregnancy.

## Supplementary Information

**Additional file 1: Supplementary Table S1.** Median of key characteristics of women included in the analysis.

## Data Availability

The data used is freely available to the public through the link https://dhsprogram.com/data/available-datasets.cfm.

## Abbreviations

DHS: Demographic and Health Survey
OR: Odds ratio
SSA: Sub-Saharan Africa
