# Supplementary material for: Proximate and distal factors associated with the stall in the decline of adolescent pregnancy in Uganda
Source: BMC Public Health. 2021 Oct 18;21:1875. doi: 10.1186/s12889-021-11403-6 (PMC8522069; doi:10.1186/s12889-021-11403-6)
Supplement: Supplementary file 1 — Additional file 1: Supplementary Table S1. Median of key characteristics of women included in the analysis. [file 12889_2021_11403_MOESM1_ESM.docx]

***Supplementary Table S1: Median of key characteristics of women included in the analysis***

| **Variable** | **Obs** | **Median** | **Inter Quartile Range (IQR)** |
| --- | --- | --- | --- |
| Age | 9937 | 17 | (16; 18) |
| Age at first Sex | 4485 | 15 | (14;17) |
| Age at first marriage | 2399 | 16 | (16;18) |
| Age at first Birth | 1988 | 17 | (15;17) |
| Years of schooling | 9936 | 6 | (14;17) |
